# Assessing the relative impacts and economic costs of Japanese knotweed management methods

**DOI:** 10.1038/s41598-023-30366-9

**Published:** 2023-03-17

**Authors:** Sophie Hocking, Trisha Toop, Daniel Jones, Ian Graham, Daniel Eastwood

**Affiliations:** 1 Department of Biosciences, Swansea University, Singleton Park, Swansea, SA2 8PP UK; 2 Agri-EPI Centre, Poultry Lane, Edgmond, Newport, TF10 8JZ England, UK; 3 Harper Adams University, Poultry Lane, Edgmond, Newport, TF10 8NB England, UK; 4 Advanced Invasives Ltd., Sophia House, 28 Cathedral Road, Cardiff, CF11 9LJ UK; 5 Complete Weed Control Ltd., Unit 16, Hurworth Road, Newton Aycliffe, DL5 6UD UK

**Keywords:** Environmental impact, Ecology

## Abstract

Sustainable land management encompasses a range of activity that balance land use requirements with wider conservation and ecosystem impact considerations. Perennial invasive alien plants (IAPs), such as Japanese knotweed, cause severe ecological and socio-economic impacts, and methods to control their spread also come at a cost. Synthetic herbicides are generally viewed as less sustainable and more ecologically damaging than alternative approaches. Here we used a comparative Life Cycle Assessment to evaluate the sustainability of herbicide-based management approaches and physical alternatives, using a large-scale Japanese knotweed field study as a model IAP system. Glyphosate-based methods elicited the lowest environmental impacts and economic costs during production. Geomembrane covering and integrated physiochemical methods were the costliest and imposed the greatest impacts. We discuss the costs and benefits of chemical and physical approaches for the sustainable management of invaded land and question how sustainable environmental stewardship is defined for the control of IAPs.

## Introduction

As global focus on environmental sustainability rises, herbicides have been scrutinised due to their environmental, ecological and social impacts^1,2^. Herbicide application plays an important role in managing invasive alien plants (IAPs)^3^, which themselves impose negative impacts^4–6^. However, with increasing demand for sustainable solutions, alternative management methods are postulated to impose less damage^7^. The viability of biocontrol agents has been investigated^8,9^, the use of root exudates and other natural alternatives^2^ and physical management methods such as mowing^10^, excavation^11^, covering (reviewed by Dusz et al.^12^), and electrical treatment^13^ are also gaining interest to ensure alignment with sustainable management goals.

Despite increasing focus on novel management solutions, evidence of the relative impacts of these different approaches is limited. Moreover, impact assessments often focus on implications following application; this represents just one stage in the life cycle of IAP management methods. Raw material extraction, production, formulation, packaging, storage, transport and use are intrinsic processes of any approach used for IAP control. If these stages are omitted from assessment, prioritisation of IAP treatment options may become skewed towards those that exhibit low impacts in the use and post-use phase, irrespective of their overall environmental risk. Regardless of motivation to constrain herbicide use, chemical methods are particularly important for some invasive plants^3^. Japanese knotweed (*Reynoutria japonica* var. *japonica*) is a well-known example of the difficulties associated with perennial IAP management. Complications in managing knotweed arise from its plasticity in environmental tolerance^14,15^, resilience to physical disturbance^16,17^, vegetative dispersal capabilities^18^, and extensive energy storage in rhizomes^19^. This IAP negatively impacts native ecosystems, reducing biodiversity and altering provision of ecosystem services^20,21^. The perceived threat of property damage resulting from knotweed infestation has also impacted mortgage lending and housing valuation^22^. Sustainable management is therefore imperative.

Numerous treatment methods have been proposed for Japanese knotweed^8,11,23^ with varying degrees of success. Physical methods (including covering, cutting, burning, digging and encapsulation) are of particular interest as they are considered more efficient for development sites. However, these methods are labour intensive, expensive and some (particularly cutting) may exacerbate knotweed dispersal^24^. Biological control has also been researched extensively as an environmentally friendly option, albeit with limited evidence of success to-date^25^. Chemical approaches employing glyphosate are considered the most successful for knotweed management^23,24^. Nevertheless, there are negative social perceptions of herbicides due to concerns around impacts to biodiversity and human health. This increases the risk of deregulation, jeopardising effective knotweed management^26^ and wider IAP control; particularly given that rates of biological invasion have not yet reached saturation^27^.

There is a trade-off between control efficacy and the impacts of management^28^; both facets of our wider responsibility to mitigate negative anthropogenic effects on the environment. Understanding the consequences of this relies on long-term data collection at a relevant scale, a key gap in invasion science^29^. To avoid shifting burdens, management options must be informed by control efficacy and impacts to the environment and human health^28,30^. Impacts across the life cycles of treatment methods should be considered to determine the true sustainability of IAP management and identify priorities that align with national and global commitments to sustainability and natural resource management^31^.

Economic costs are also pertinent to the selection of IAP management strategies. The global annual cost of invasive species management is estimated to be US$26.8 billion^32^. Japanese knotweed has been calculated to cost £165,609,000 per year in the UK^4^. Current estimates of costs are primarily associated with knotweed management at development sites, road and rail networks, in private land or gardens, and in semi-natural habitats. Indirect costs associated with knotweed include legal advice and action, and property devaluation. Thus, the economic costs of knotweed impact a variety of sectors as well as the general public and local authorities. Comparing the cost of management methods can inform viability and prioritisation of methods to ensure effective resource use, particularly relevant when managing at scale.

Public perception is increasingly important for the reporting and implementation of sustainable invasive species management^33^. Value systems, bias, impacts of invasive species and economic interests are key factors influencing perception and therefore support of management approaches^34,35^. Recent research shows disparity between the views of nature experts, users and the general public when it comes to acceptance of invasive species management methods^36^, highlighting the importance of stakeholder engagement in sustainability reporting and robust scientific evaluation of management approaches. By considering the impacts and costs of products used in IAP management across whole life cycles, the sustainability of IAP management methods can be better informed, fostering meaningful alignment with sustainability goals. To determine the wider sustainability of IAP management methods, this study conducted a comparative Life Cycle Assessment (LCA) of Japanese knotweed treatment methods using a long-term, large-scale field trial (Jones et al.^23^) as a case study. While the products used for invasive plant management have likely been subject to LCA during their formulation, this information is unavailable to the public and there is little comparison of such products in the context of long-term, field-relevant model systems that represent realistic environmental scenarios. This study therefore aims to contribute to this knowledge gap by assessing the environmental impacts of Japanese knotweed control strategies using LCA, and evaluating the relative economic costs to ensure meaningful alignment with sustainability objectives in weed management.

## Methods

### Goal of the life cycle assessment

The goal of this study is to assess and compare the impacts of seven Japanese knotweed management methods during the production phase. The management methods used in this study are based on a long-term study of Japanese knotweed management by Jones et al.^23^ and involve chemical treatment using different timings and application rates of glyphosate, picloram and 2,4-D, integrateing physiochemical methods including digging and geomembrane covering. Full details are found in Table 1. Environmental impacts were modelled and evaluated using the impact indicators provided in the ReCiPe impact assessment method at midpoint (18 categories) and endpoint (3 categories) level^37^ (Supplementary Table S1). These indicators were used to provide a comprehensive picture on the relative impacts of each treatment. The economic costs to implement each method were also compared across treatments to collectively provide a basis for the evaluation of the implications and practicality of these methods for large-scale knotweed management.

**Table 1:** Japanese knotweed control treatment groups of interest for LCA (Adapted from Jones, 2015 and Jones et al.^23^). Treatment codes refer to the herbicide active ingredient (G = glyphosate, D = 2,4-D, P = picloram), application rate, application method (F = foliar spray, Sl = soil spray, St = stem injection, Cov = covering), and time of year applied (S = spring, A = autumn). Specific timing of seasonal application: spring = April-June; autumn = September–November.

Treatment code	Description	Application rate (kg AE ha^−1^)
G^3.60, F, A^	Annual autumn foliar spray; Glyfos ProActive®	3.60
G^2.16, F, S+A^	Summer	2.16
	Glyphosate (Glyfos ProActive®) foliar spray	
	Autumn	
	Glyphosate (Glyfos ProActive®) foliar spray	
D^2.80^ + G^2.16; F, S+A^	Spring	
	2,4-D (Depitox®) foliar spray	2.80
	Glyphosate (Glyfos ProActive®) foliar spray	2.16
	Autumn	
	2,4-D (Depitox®) foliar spray	
	Glyphosate (Glyfos ProActive®) foliar spray	
P^2.69, F+Sl, S^ + G^3.60, F, A^	Early spring	
	Picloram (Tordon 22 k®) foliar and soil spray	2.69
	Autumn	
	Glyphosate (Glyfos ProActive®) foliar spray	3.60
G^65.00, St, A^	Autumn	
	Glyphosate (Glyfos ProActive®^)^ stem injection (3–5 ml undiluted herbicide per stem)	65.00
D^S^ + G^3.60, F, A^	Spring	
	Digging and turning of knotweed rhizome	
	Autumn	
	Glyphosate (Glyfos ProActive®) foliar spray	3.60
D + P^2.69, F+Sl, S^ + G^3.60,F, A^	Spring	
	Digging and turning of knotweed rhizome	
	Picloram (Tordon 22 K®) foliar and soil spray	2.69
	Autumn	
	Glyphosate (Glyfos ProActive®) foliar spray	3.60
Mem^Cov^	All year	
	Cover with Viqueen® 300 µm (1200 gauge) HDPE geomembrane	N/A
	All year	
	Hand pull emergent knotweed	

### Life cycle assessment scope

This study used a large-scale Japanese knotweed control field trial based in South Wales, UK, as a model system^23^. While the aim of Jones et al.^23^ was to assess knotweed treatment efficacy, data was retained on the materials and products used and time consumption per treatment. This provides data to assess the impacts of knotweed management methods at a field-relevant scale. All treatments applied in Jones et al.^23^ were used according to manufacturer’s guidelines and are therefore assumed to be representative of treatment methods widely used for invasive plant management in Europe and North America. Treatments were assessed using 225 m^2^ field plots, with each treatment replicated in triplicate, allowing most treatments to be assessed over a total area of 675 m^2^, except the covering treatment, which consisted of a single plot (225 m^2^). As this model system assessed treatment methods over multiple years, data was available to evaluate the long-term impacts of each management approach. Pulling, burning and digging of Japanese knotweed was not assessed in this study as these approaches were considered too costly, labour intensive and could also increase risk of knotweed dispersal^23^.

### Treatments selected for LCA

Treatment groups selected for LCA were a sub-set of treatments tested by Jones et al.^23^ and were chosen as representative of chemical, physiochemical and physical management methods based on efficacy and current industry recommendations (Table 1).

### Description of treatment methods used in the LCA

Annual herbicide application data was converted to total herbicide use per hectare (ha) measured as (L ha^−1^) and active ingredient acid equivalent (AE) per hectare (kg AE ha^−1^) (Supplementary Table S2). The constituents of each treatment, authorised application rates and actual application rates used by Jones et al. are summarised in Supplementary Table S2.

Herbicide use for treatment P^2.69, F+SL, S^ + G^3.60, F, A^ (Early spring Picloram (Tordon 22 k®) foliar and soil spray and Autumn Glyphosate (Glyfos ProActive®) foliar spray) could not be measured from 2015 onwards as the use of picloram (the active ingredient in Tordon 22 K®) was restricted in the EU. Therefore, the actual amount of herbicide used was assessed, as well as projected values of total herbicide used if the use of picloram had not been restricted. Since the application rate for Tordon 22 K® would have remained approximately consistent each year, projected values were obtained by multiplying the amount of herbicide used in year 2 by 3; total application rate therefore included recorded application rates for years 1 and 2 and projected application rates for years 3 to 5. This approach was also applied to treatment D + P^2.69, F+Sl, S^ + G^3.60,F, A^ (picloram and glyphosate application).

#### Chemical knotweed treatment information

As detailed in Jones et al.^23^, herbicides were applied with dye and adjuvant (Topfilm; 1.2 L ha^−1^) using a knapsack sprayer fitted with a 0.75–1.5 m telescopic lance and flat fan nozzle. Prior to initial soil application of picloram (P^2.69, F+SL^, S + G^3.60, F, A^), aboveground Japanese knotweed stem and leaf litter was cleared to facilitate uniform soil coverage and enable herbicide transport to emerging shoots and the rhizome. For stem injection application (G^65.00, ST, A^), in autumn during initial treatment, individual stems were injected with undiluted glyphosate (3–5 ml injection volume; equivalent to 65.00 kg AE ha^−1^). Adjuvant was not included for stem injection. In subsequent years, foliar spray application of glyphosate (3.60 kg AE ha^−1^) was undertaken in autumn.

#### Integrated physical and chemical methods

For cutting and foliar spray application of glyphosate in autumn (D^S^ + G^3.60, F, A^), Japanese knotweed was cut in the summer using a Stihl FS-450 Professional 2.1 kW clearing saw. Foliar spray application of glyphosate (3.60 kg AE ha^−1^) was performed in autumn, and repeated in subsequent years. Excavation (D^S^ + G^3.60, F, A^ and D + P^2.69, F+Sl, S^ + G^3.60, F, A^) was conducted in spring using a JCB 3CX backhoe loader to a depth of 2.5 m, with rhizome material sorted and concentrated at the soil surface by the operator. This was followed by soil spray application of picloram (Tordon; 2.69 kg AE ha^−1^) in spring for P^2.69, F+SL, S^ + G^3.60,F, A^. Both treatments D^S^ + G^3.60, F, A^ and D + P^2.69, F+Sl, S^ + G^3.60,F, A^ received foliar spray application of glyphosate (3.60 kg AE ha^−1^) in autumn. Excavation was only performed in the first year of treatment, though soil and foliar spray application of herbicides continued in following years.

#### Physical knotweed management

Covering (Mem^Cov^) was the only physical management method tested by Jones et al.^23^. Knotweed litter was flattened and left in-situ prior to the emergence of new growth. High-density polyethylene (HDPE) geomembrane (Visqueen® 300 µm 1200 gauge) was placed over the treatment area in early spring and kept in position for the duration of the experiment. Knotweed growth beneath the membrane was flattened and visible emergence round the covering was hand pulled and left under the membrane.

### System boundaries and functional unit

**Figure 1:**
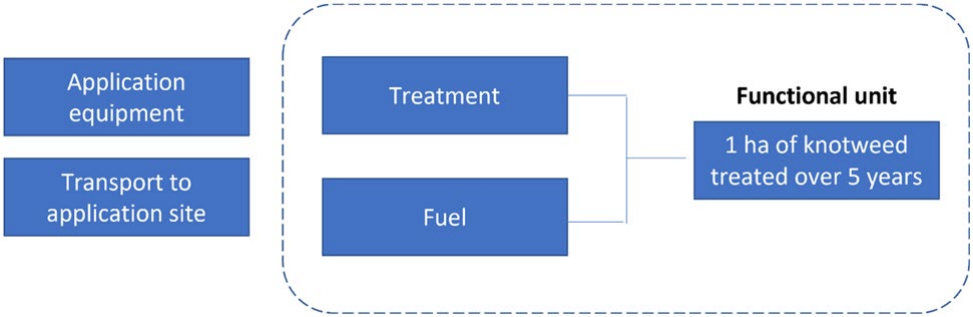
General system boundaries for this comparative LCA. Composition of each treatment is detailed in Table 2.

This LCA covers the production stage of seven Japanese knotweed treatment methods (Table 1; Fig. 1). The system boundary includes active ingredient manufacturing with material and energy inputs, production of inert ingredients and mixing, blending and dilution of herbicide active ingredient with inert ingredient to create herbicide products. Production and transport of co-formulates (i.e., tallow amine) and packaging of herbicides are also included within the system boundaries. Herbicide application equipment (e.g. knapsack sprayers) were omitted from this study as they were common to all chemical treatment methods and it was not considered to directly contribute to impacts in knotweed management as they are re-usable. Spray additives (e.g. Topfilm®) were also omitted from this LCA as there is insufficient data relating to their production.

The functional unit in this study is the application of 1 ha of Japanese knotweed control treatments over a 5-year period. This functional unit was chosen as it reflects established knowledge of practical treatment of aboveground knotweed growth at a field-relevant spatial scale^23^.

### Data inputs

Material inputs for each treatment method (converted to kg ha^−1^) were calculated from long-term records maintained as part of on-going Japanese knotweed control field trials (Table 2). Tallow amine ethoxylate is a co-formulant commonly used in glyphosate-based formulations that is present as 9% w/w in Glyfos ProActive®. The ratio of glyphosate to tallow amine was calculated to be 4.58:1 from the product labels of herbicides used in Jones et al.^23^. This was used to calculate the amount of tallow amine used per treatment group (Eq. (1))

**Table 2:** Data inputs for each treatment method.

Treatment group	Component(s)	Mass (kg) used per ha
G^3.60, F, A^	Glyphosate	16.9
	Tallow amine	3.69
G^2.16, F, S+A^	Glyphosate	18.43
	Tallow amine	4.03
D^2.80^ + G^2.16; F, S+A^	Glyphosate	17.25
	Tallow amine	3.77
	2,4-D	16.3
P^2.69, F+Sl, S^ + G^3.60, F, A^	Glyphosate	17.15
	Tallow amine	3.75
	Picloram	9.74
	Petrol, 2 stroke blend	220
P^2.69, F+Sl, S^ + G^3.60, F, A(projected)^	Glyphosate	17.15
	Tallow amine	3.75
	Picloram	14.62
	Petrol, 2 stroke blend	220
G^65.00, St, A^	Glyphosate	70.91
	Tallow amine	15.49
D^S^ + G^3.60, F, A^	Glyphosate	25.31
	Tallow amine	5.53
	Diesel	1540.76
D + P^2.69, F+Sl, S^ + G^3.60,F, A^	Glyphosate	21.62
	Tallow amine	4.72
	Picloram	25.19
	Diesel	1540.76
D + P^2.69, F+Sl, S^ + G^3.60,F, A(projected)^	Glyphosate	21.62
	Tallow amine	4.72
	Picloram	30.26
	Diesel	1540.76
Mem^Cov^	HDPE geomembrane	2650

1 $$Tallow\,amine \left(kg\right)=\frac{Total\,kg\,glyphosate\,used}{4.58}$$

Petrol and diesel inputs were related to the use of machinery for digging and cutting vegetation in physiochemical treatments (Table 2).

Data from Life Cycle Inventory (LCI) databases Agri-footprint (Blonk Sustainability, Netherlands) and EcoInvent 3 (Ecoinvent, Switzerland) were used for upstream production processes (Supplementary Table S3). These databases can be used to model environmental impacts based on robust, quantitative data. Compatibility and consistent methodological approaches were checked across the databases before use to ensure the validity of the results produced. Specific materials and processes used in SimaPro are shown in Supplementary Table S3.

### LCA impact assessment (LCIA)

The impacts of each treatment method were compared using the ReCiPe 2016 Midpoint and Endpoint LCIA method^37^ in SimaPro 9.0.0.48 PhD (Pré Sustainability, Netherlands). The hierarchist (H) impact assessment method was used at both midpoint (problem-oriented, 18 impact categories) and endpoint (damage-oriented, 3 impact categories) assessment levels. The hierarchist approach is based on scientific consensus of appropriate timescales and plausibility of impact mechanisms^37^; the timescale adopted in evaluating impacts using this approach is 100 years. Sensitivity analysis was conducted to determine whether impact assessment method influenced the results. Values of impacts calculated for the eight knotweed management approaches assessed across three common impact categories calculated by ReCiPe 2016, ILCD 2011 midpoint + V1.10 and EF 3 were found to be non-normally distributed and were compared using a one-sided Kruskal–Wallis test to determine significant differences. Statistics and graphical presentation were conducted in R 3.4.3^38^ using the package ggplot2^39^.

### Economic evaluation

The economic evaluation focuses on the costs of implementing Japanese knotweed treatments. Costs were assessed under the functional unit of the LCA (i.e., they are evaluated as £ ha^−1^ 5 yrs), though production costs were not calculated due to the number of materials used in herbicide production. The economic evaluation included material costs, time spent per treatment, fuel costs for implementing treatments and labour costs. Inflation and higher fuel costs (e.g. travel to site) were excluded as these costs commonly affect all treatments and therefore are not expected to influence interpretation of relative costs across treatment methods.

#### Data inputs

Economic evaluation of the treatment methods included prices of packaged products (GBP£) collected from Agrigem Ltd (Supplementary Table S4). The price of Monsanto Amenity Glyphosate was used in lieu of Glyfos Proactive® as this product was withdrawn from UK use in 2018. The mean price of Icade® and Synero® was used as a proxy for Tordon 22 K which was deregulated in 2015, as these products most closely resemble Tordon 22 K. The price of Visqueen HDPE geomembrane was obtained from the Visqueen website. Fuel costs are omitted here as it is included in costs of machine operation. Data for time consumption (hours) per treatment was collated from field records and converted to time (hrs) ha^−1^ per year (Table 3).

**Table 3:** Data inputs for time consumption per year for herbicide application (obtained from records from study system).

Treatment	Time consumption (hrs) ha^−1^ year					Total
	Year 1	Year 2	Year 3	Year 4	Year 5	
G^3.60, F, A^	29.7	22.22	22.22	22.22	8.47	104.76
G^2.16, F, S+A^	51.85	40.74	40.74	44.44	44.44	222.21
D^2.80^ + G^2.16; F, S+A^	59.26	44.44	31.11	31.11	31.11	197.03
P^2.69, F+Sl, S^ + G^3.60, F, A^	74.07	59.26	59.26	22.22	8.47	223.28
P^2.69, F+Sl, S^ + G^3.60, F, A (projected)^	74.07	59.26	59.26	59.26	45.51	297.36
G^65.00, St, A^	140.74	22.22	22.22	22.22	8.47	513.23
D^S^ + G^3.60, F, A^	18.52	14.81	14.81	7.41	14.81	70.36
D + P^2.69, F+Sl, S^ + G^3.60,F, A^	33.33	37.03	37.03	7.41	14.81	129.61
D + P^2.69, F+Sl, S^ + G^3.60,F, A(projected)^	33.33	37.03	37.03	22.22	29.62	159.23
Mem^Cov^	N/A	N/A	N/A	N/A	N/A	N/A

The time spent on physical components of Japanese knotweed treatments was also recorded and collated (Table 4).

**Table 4:** Data inputs for physical components of Japanese knotweed treatments. N/A = not applicable as treatments did not include physical management methods.

Treatment	Description	Time (hrs) ha^−1^
G^3.60, F, A^	N/A	N/A
G^2.16, F, S+A^	N/A	N/A
D^2.80^ + G^2.16; F, S+A^	N/A	N/A
P^2.69, F+Sl, S^ + G^3.60, F, A^	Stem and leaf clearance	148.15
P^2.69, F+Sl, S^ + G^3.60, F, A (projected)^	Stem and leaf clearance	148.15
G^65.00, St, A^	N/A	N/A
D^S^ + G^3.60, F, A^	Excavation	133.33
D + P^2.69, F+Sl, S^ + G^3.60,F, A^	Excavation	133.33
D + P^2.69, F+Sl, S^ + G^3.60,F, A(projected)^	Excavation	133.33
Mem^Cov^	Installation of geomembrane and hand-pulling emergent knotweed	2666.66

Time consumption data was used to calculate labour costs based on representative salaries for weed control practitioners, confirmed by specialist amenity weed management contractor Complete Weed Control Ltd. For excavation, costs of machine hire and labour are combined and based on a 10-h working day (information obtained from Marlay Project Management Ltd; Supplementary Table S5).

Material costs, time spent per treatment and labour costs were calculated per ha per treatment to align with the functional unit. Although this study spans 5 years (per the functional unit), inflationary costs and cost fluctuations are not included.

### Integration of LCA with economic evaluation

The economic costs of endpoint environmental impacts calculated by LCA can be monetised using conversion factors. As environmental impacts inherently incur economic costs, the total costs per treatment were calculated, to better inform the economic impacts of Japanese knotweed treatment methods. This could contribute to informing costs that can be mitigated during the development of plant protection products and approaches. There are three endpoint impact categories: human health (disability-adjusted life years, DALYs), ecosystems (lost species-year) and resource use (US dollars). The conversion factors used in this study follow Ögmundarson et al.^40^ where the conversion factor for human health is 100,000 USD/DALY, and 65,000 USD/species.yr for impacts to ecosystems.

Costs of environmental impacts are calculated using the following Eq. (2)^41^:

2 $${C}_{LCA}= \sum_{i=1}^{3}{m}_{i}{ED}_{i}$$

where ${ED}_{i}$ is the environmental impact assessment results and ${m}_{i}$ is the conversion factor for the *i*th damage category of the LCIA^41^. Once converted from impacts to costs (USD), this was converted to GBP for consistency. The total costs per treatment were then calculated using Eq. (3)^41^:

3 $${F}_{c}= {C}_{LCA}+ {C}_{e}$$

where ${F}_{c}$ is the total cost per treatment, ${C}_{LCA}$ is the cost of environmental impacts and ${C}_{e}$ is the economic cost of implementing each treatment.

## Results

### LCA midpoint and endpoint results

#### Evaluating impacts of knotweed management approaches at midpoint level

**Figure 2:**
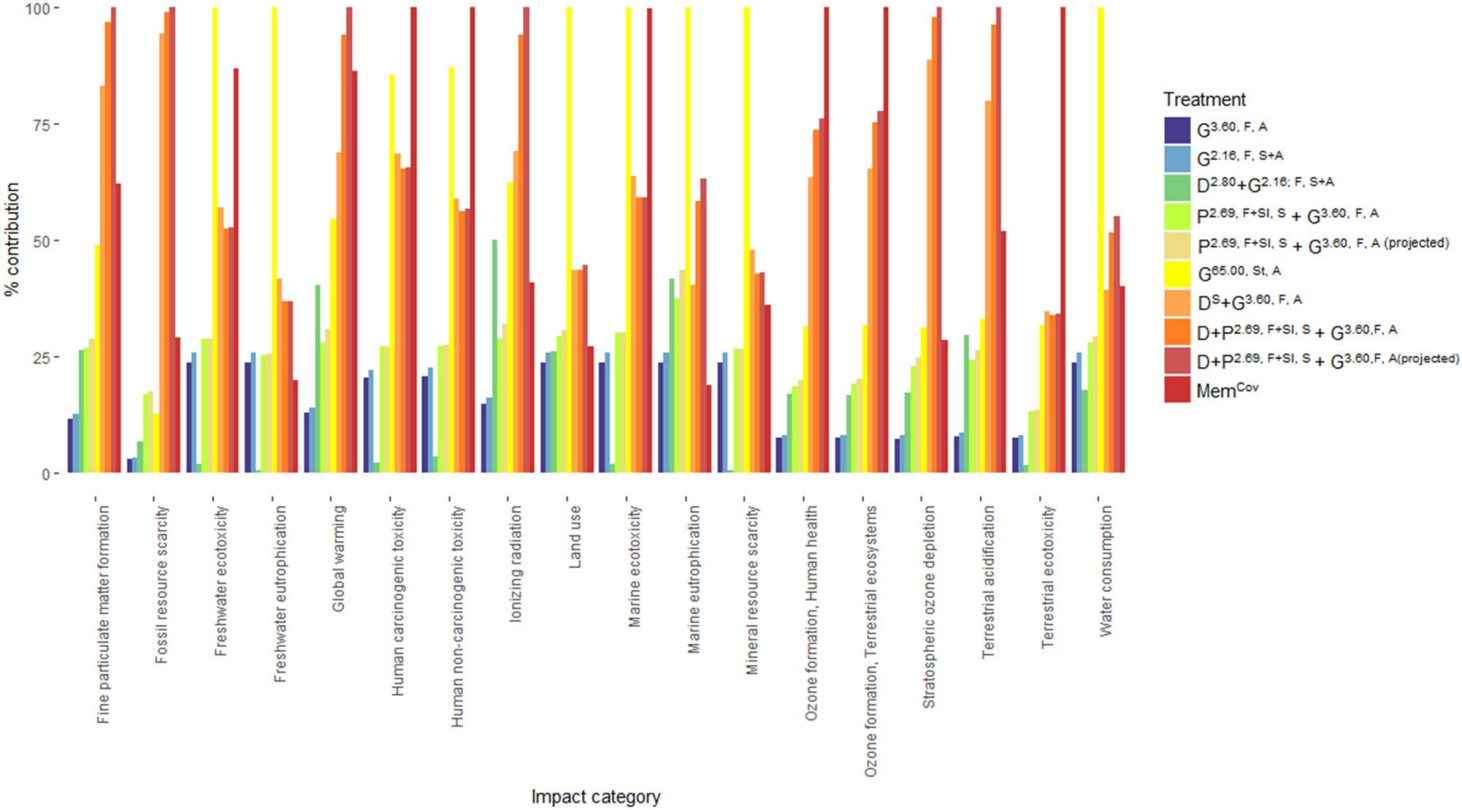
Relative contribution (%) of Japanese knotweed treatment methods to midpoint impact categories using the ReCiPe assessment method. See Table 1 for description of treatments.

Treatments involving physical methods (D + P^2.69, F+Sl, S^ + G^3.60,F, A^, D + P^2.69, F+Sl, S^ + G^3.60,F, A(projected)^ and Mem^Cov^) showed the highest impacts in 11 out of 18 impact categories compared to chemical methods (namely, stem injection, G^65.00, St, A^) which made the highest contribution to six categories (Supplementary Table S6, Fig. 2). High Density Polyethylene (HDPE) Geomembrane covering of Japanese knotweed (Mem^Cov^) showed the greatest contribution to six out of 18 impact categories (Supplementary Table S6, Fig. 2). Stem injection and geomembrane covering showed the highest contribution to marine ecotoxicity, indicating that the amount of product used in each treatment is an important factor influencing production impacts.

Stem injection (G^65.00, St, A^) contributed the most to freshwater ecotoxicity, freshwater eutrophication, land use, marine eutrophication, mineral resource scarcity and water consumption (Supplementary Table S6, Fig. 2). Geomembrane covering (Mem^Cov^) exhibited the greatest impacts to human carcinogenic and non-carcinogenic toxicity, ozone formation affecting human health and terrestrial ecosystems and terrestrial ecotoxicity (Supplementary Table S6, Fig. 2).

Excavation integrated with spraying (D + P^2.69, F+Sl, S^ + G^3.60,F, A^ and D + P^2.69, F+Sl, S^ + G^3.60,F, A(projected)^) contributed the greatest to fine particulate matter emissions, fossil resource scarcity, global warming, ionizing radiation, stratospheric ozone depletion and terrestrial acidification (Supplementary Table S6; Fig. 2). Digging and turning of knotweed integrated with glyphosate spray (D^S^ + G^3.60, F, A^) was frequently the next greatest contributor to these categories.

No treatment involving foliar herbicide application alone made the greatest contribution to any category (Supplementary Table S6; Fig. 2). Glyphosate foliar spray (G^3.60, F, A^ and G^2.16, F, S+A^) had the lowest impacts across 10 impact categories (Supplementary Table S6, Fig. 2). The impacts of bi-annual glyphosate foliar spray (G^2.16, F, S+A^) were 1.1 × greater than single annual glyphosate application (G^3.60, F, A^) (Supplementary Table S6).

Integrated picloram and glyphosate application (P^2.69, F+Sl, S^ + G^3.60, F, A^ and P^2.69, F+Sl, S^ + G^3.60, F, A(projected)^) exhibited consistently higher impacts than glyphosate application alone (G^3.60, F, A^ and G^2.16, F, S+A^) (Supplementary Table S6, Fig. 2). Integrated 2,4-D and glyphosate foliar application (D^2.80^ + G^2.16; F, S+A^) exhibited greater impacts to global warming, ionizing radiation, and terrestrial acidification than glyphosate and picloram application (P^2.69, F+Sl, S^ + G^3.60, F, A^ and P^2.69, F+Sl, S^ + G^3.60, F, A(projected)^) and glyphosate application alone. This suggests 2,4-D production has greater impacts to these categories than other herbicides. However, D^2.80^ + G^2.16; F, S+A^ had the lowest impacts to freshwater ecotoxicity and eutrophication, human carcinogenic and non-carcinogenic toxicity, marine ecotoxicity, mineral resource scarcity, terrestrial ecotoxicity and water consumption (Supplementary Table S6, Fig. 2).

#### Evaluating use of impact assessment method: results of sensitivity analysis

**Figure 3:**
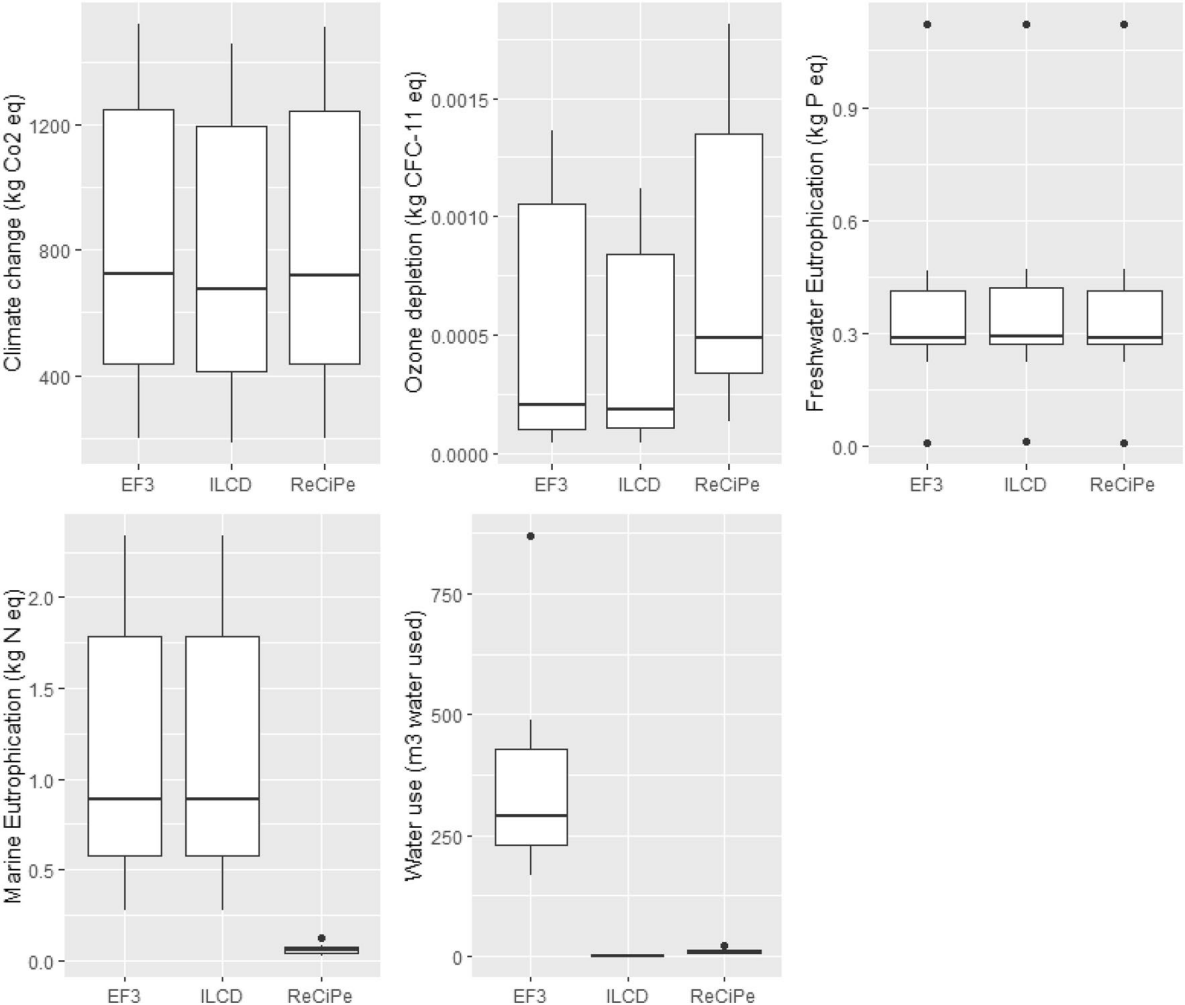
Sensitivity analysis of common impact categories (n = 3) across ReCiPe, ILCD and EF 3.0 impact assessment methods for the eight knotweed management approaches assessed in this LCA. In the box plots the center line = median; box limits = upper and lower quartiles; whiskers = 1.5 × interquartile range; points = outliers.

The results for climate change, ozone depletion and freshwater eutrophication were consistent across impact assessment methods (Fig. 3). Impacts to marine eutrophication were significantly lower for ReCiPe (H = 19.4, *df* = 2, *p* < 0.005), and calculations for water use were significantly greater using the EF 3 assessment method (H = 25.8, *df* = 2, *p* < 0.005) (Fig. 3). This suggests impacts to marine eutrophication and water use may be underestimated using the ReCiPe method.

#### Evaluating endpoint impacts of knotweed management

**Figure 4:**
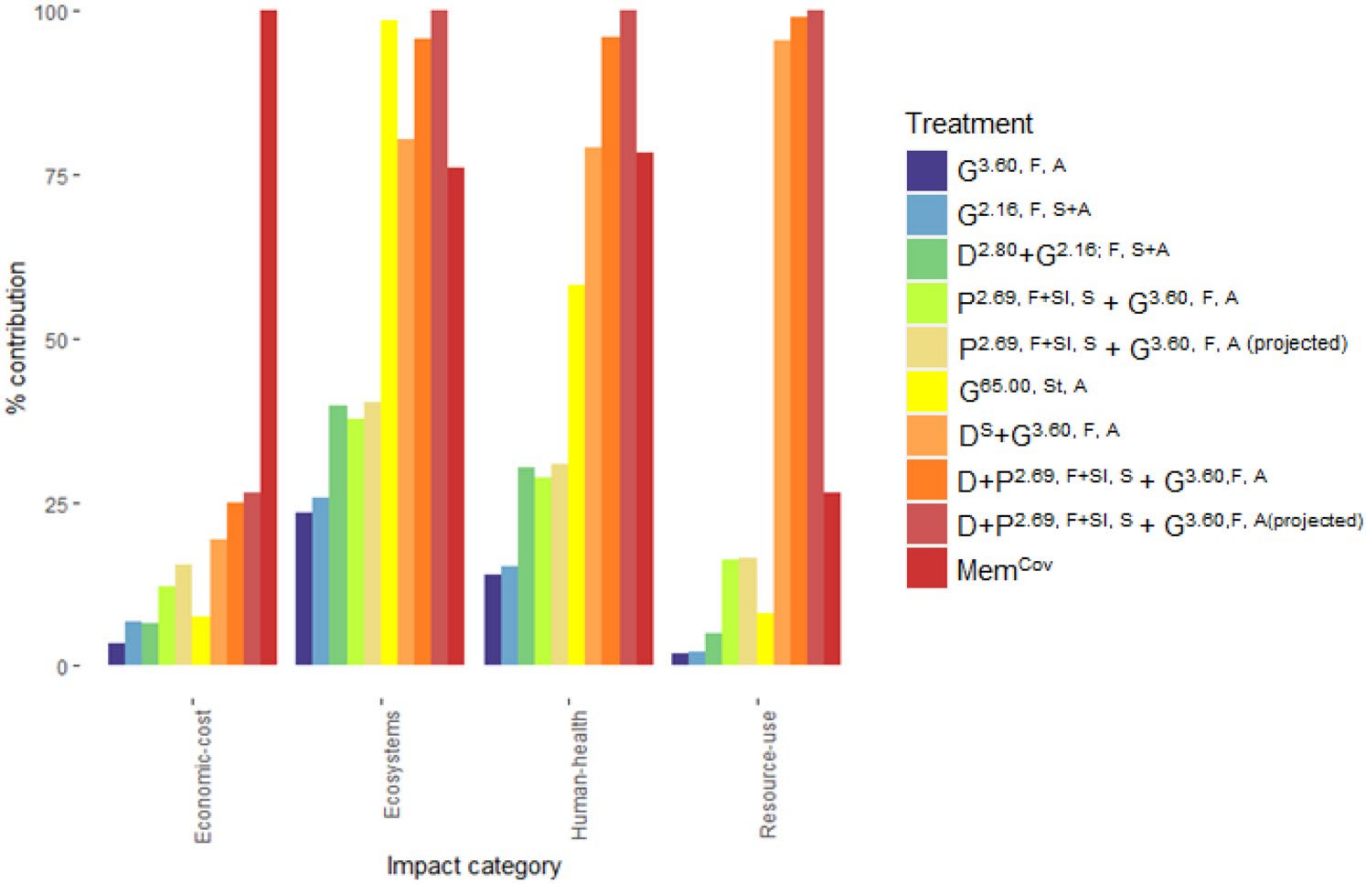
Relative impacts and economic cost of Japanese knotweed treatment methods at endpoint level. See Table 1 for description of treatments.

Excavation followed by picloram and glyphosate foliar spray (D + P^2.69, F+Sl, S^ + G^3.60, F, A(projected)^ and (D + P^2.69, F+Sl, S^ + G^3.60, F, A^) revealed the greatest impacts in most endpoint categories (Supplementary Table S7, Fig. 4). Glyphosate stem injection (G^65.00, St, A^) and projected physiochemical methods (D + ^P2.69, F+Sl, S^ + G^3.60,F, A(proj)^) exhibited the two greatest impacts to ecosystems. Geomembrane covering (Mem^Cov^) incurred the greatest economic impact (Supplementary Table S7, Fig. 4). Glyphosate foliar spray treatments (G^3.60, F, A^ and G^2.16, F, S+A^) had the lowest impacts to most endpoint categories. Chemical treatments involving picloram (P^2.69, F+Sl, S^ + G^3.60, F, A^) had greater impacts to resource use than glyphosate stem injection, highlighting differences in the production of these herbicides.

#### Economic evaluation of knotweed management approaches

Geomembrane covering (Mem^Cov^) incurred the greatest total costs (costs to implement treatment and cost of environmental impacts), followed by physiochemical methods (D + P^2.69, F+Sl, S^ + G^3.60,F, A(projected^; D + P^2.69, F+Sl, S^ + G^3.60,F, A^, and D^S^ + G^3.60, F, A^,), and treatment involving picloram (P^2.69, F+Sl, S^ + G^3.60, F, A(projected)^; P^2.69, F+Sl, S^ + G^3.60, F, A^) (Supplementary Table S7, Fig. 4). Glyphosate treatments incurred the lowest costs (G^3.60, F, A^,; G^2.16, F, S+A^; D^2.80^ + G^2.16; F, S+A^ and G^65.00, St, A^; Supplementary Table S7, Fig. 4). Costs to implement treatment methods (including material and labour) accounted for 95.5% ± 2.8 of costs per treatment, mainly made up by labour costs due to the time taken to implement each treatment.

#### Comparison of time consumption across treatment methods

**Figure 5:**
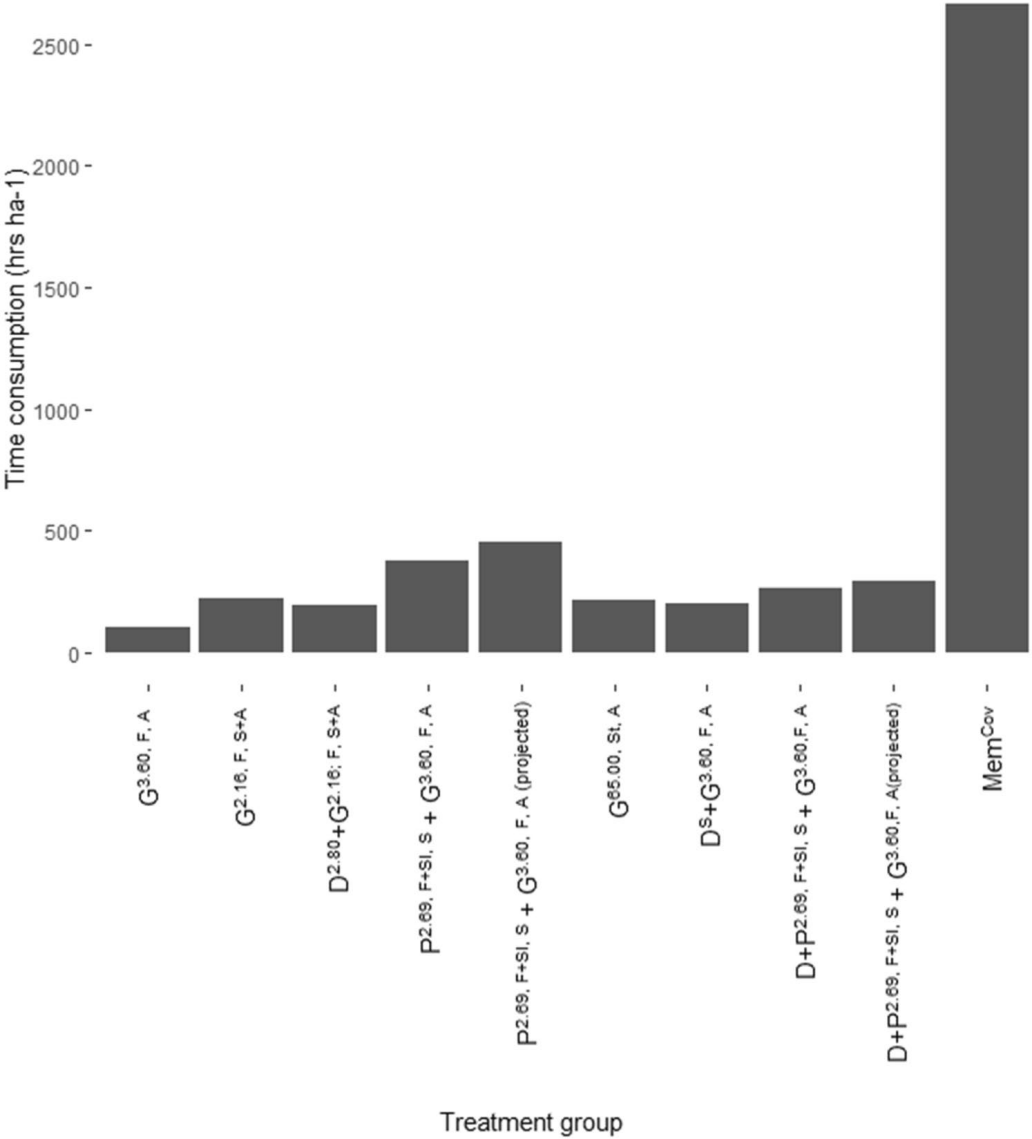
Time taken to implement each treatment (collated from records of study system^23^). See Table 1 for description of treatments.

Geomembrane covering (Mem^Cov^) had the greatest time consumption (2,666.7 h ha^−1^; Fig. 5) due to the time taken to install geomembrane and repeated hand-pulling of emergent Japanese knotweed emerging around the membrane. Due to the need for vegetation clearance, treatments involving picloram and glyphosate spray (P^2.69, F+Sl, S^ + G^3.60, F, A^ and P^2.69, F+Sl, S^ + G^3.60, F, A(projected)^) exhibited the second-highest time consumption (378.8 and 452.9 h ha^−1^, respectively; Fig. 5).

## Discussion

The aim of this study was to compare the sustainability of Japanese knotweed treatment approaches at a field-relevant scale using empirical data from a large-scale, long-term project. This information is intended to inform management decisions and contribute to our understanding of the relative impacts of physical and chemical management methods.

The relative contribution to impact categories under the ReCiPe hierarchist method and economic costs of implementing these treatments were used to determine the most economically and environmentally viable options. The modelled impacts are based on the hierarchist approach to LCIA which uses a timescale of 100 years^30^. We found the simplest methods elicited the most favourable environmental outcomes. Foliar spray glyphosate application produced the lowest relative impacts, economic costs and time consumption as it is the most effective treatment against Japanese knotweed^23,42^. By aligning knotweed management with plant biology and ecophysiology, effective control can be achieved with relatively low doses of glyphosate^23^. Differences between foliar spray (G^3.60, F, A^ & G^2.16, F, S+A^) and stem injection (G^65.00, St, A^) arise from the larger application rate and concentration of glyphosate used in stem injection^43,44^, illustrating that environmental impacts increase with herbicide application rate. Foliar spray methods offer fewer negative impacts as the glyphosate is diluted into a spray mix applied at relatively low concentrations. As Japanese knotweed incurs some of the highest costs of all invasive species in the UK^4,45^, primarily owing to management, the results of this study may inform cost-effective management.

Differences in the impacts of integrated foliar application of 2,4-D and glyphosate (D^2.80^ + G^2.16; F, S+A^) compared to glyphosate alone highlight discrepancies in emissions resulting from 2,4-D and glyphosate production. Integrated picloram soil and glyphosate foliar application (P^2.69, F+Sl, S^ + G^3.60, F, A^ and P^2.69, F+Sl, S^ + G^3.60, F, A(projected)^) had greater midpoint and endpoint impacts than glyphosate and lower impacts than physiochemical methods (D^S^ + G^3.60, F, A^ & D + P^2.69, F+Sl, S^ + G^3.60,F, A^; Figs. 2 and 4), emphasising the simplest control methods elicited the lowest impacts. In terms of control success, picloram produces mixed results in knotweed management^46^ and is less effective than glyphosate alone^23^.

Physical and integrated physiochemical methods exhibited the greatest negative environmental impacts in this study (Figs. 2 and 4). The impacts of High-Density Polyethylene (HDPE) geomembrane covering of Japanese knotweed (Mem^Cov^) arise from crude oil extraction, distillation, cracking and extrusion processes involved in plastic manufacture which are resource and energy intensive and produce substantial emissions^47–49^. This is evident from the LCA results for Mem^Cov^ (Supplementary Table S6). Geomembrane covering also had the greatest total economic costs (Fig. 4), owing to the costs and time consumption associated with implementing this treatment (Fig. 5). This is highlighted by Rask et al.^50^ who found less effective physical methods require more intense treatment to achieve levels of control equivalent to herbicide application. In this model study, covering was an ineffective management strategy against Japanese knotweed at field scale^23^.

The impacts of integrated physiochemical Japanese knotweed management (D^S^ + G^3.60, F, A^ and D + P^2.69, F+Sl, S^ + G^3.60,F, A^) arise from the use of diesel for excavation. Modelled impacts of knotweed excavation to ozone depletion resulting from crude oil extraction and distillation (associated with diesel production) and fuel combustion is consistent with literature on the impacts of soil remediation techniques^51^. Physiochemical methods also caused the greatest endpoint impacts (Fig. 4), however, time consumption was consistent with foliar spray methods (Fig. 5). While these treatments were less effective than glyphosate application alone^23^, excavation is often used where timescales may not allow for extended treatment by annual herbicide application and the cost of excavation is offset by the costs incurred through project delay (e.g. high value land development sites)^24,46^. Physical disruption by rhizome tillage may deplete plant energy reserves and accelerate control of aboveground growth^23^. However, the costs, labour requirements and need for disposal of controlled waste linked to this method are disadvantageous^24,46^. This approach may also pose a biosecurity risk through accidental dispersal of knotweed rhizome fragments.

The relative impacts of excavation versus herbicide application should be considered in relation to site-specific management objectives and available resources. Objectives prioritising biodiversity conservation, effective knotweed management and environmental sustainability may favour targeted, long-term approach. In this case, herbicides are an effective management tool^52^, though careful consideration of post-application impacts and wider ecological context is needed. Where the costs of management outweigh the impacts of Japanese knotweed, a do-nothing approach may be more sensible than employing alternative physical methods, which are less effective and elicit greater production impacts.

### Post-application impacts of Japanese knotweed management methods

The relative impacts of invasive plant management methods post-treatment are currently unknown. Research on the environmental fate of herbicides mainly focuses on agricultural settings as the primary consumer of herbicides. The presence of pesticide residues in agricultural soils is now the norm^53^; glyphosate residues have been detected in the wider environment^54,55^, food products^56^ and human populations^57–59^. A recent meta-analysis has found that cumulative exposure to glyphosate-based herbicides is associated with increased risk of non-Hodgkin lymphoma^60^; however, a prospective cohort study found that glyphosate was not statistically significantly associated with cancer at any site^61^. Other toxic effects reviewed by Mesnage et al.^62^ have also been found, though the European Food Safety Authority (EFSA) concluded evidence for its classification as “probable carcinogenic” was limited^63,64^.

Exposure to glyphosate is proposed to negatively affect hormone activity, cell and organ functioning in birds, fish and mammals exposed to high doses and chronic cumulative low doses^65–67^ and imposes selection pressure towards herbicide-tolerance in plants^65^. However, the ecological interactions of herbicide residues are complex^68^. Impacts to microbial communities are limited^69^ as microbes readily degrade residues^70^. Co-formulants in herbicide products also impose risks to human and ecological health^62,71^. This has been further confirmed by Straw et al.^72^ who concluded co-formulants in Roundup® were the cause of bee mortality, rather than the active ingredient. Conversely, Weidenmüller et al.^73^ recently found that sub-lethal exposure to glyphosate alone can reduce thermoregulation in bumblebees during periods of stress. While these studies provide valuable information on the hazards of exposure, studies under field conditions using field-relevant glyphosate concentrations would broaden our understanding.

Physical approaches to IAP management may be considered less damaging than herbicides but are associated with fossil fuel use (at large scales where hand-pulling or cutting is not feasible), contributing to a key driver of carbon emissions. Records of emissions associated with transport, disposal or encapsulation of knotweed-infested soil are limited, all of which exacerbate the impacts of physical management approaches. Conversely, herbicides may reduce the environmental impacts of agricultural weed control by minimising carbon dioxide (CO_2_) emissions associated with fuel use for physical or mechanical weed management^74^. With global commitment to reducing emissions in line with the Paris Agreement^75^, evaluation of the wider impacts of plant protection products is needed. This can provide further insight on the relative importance of environmental impacts arising from production versus use and end-of life life-cycle stages.

### Costs versus benefits in IAP management

The mismatch between production and post-application impacts of Japanese knotweed management methods emphasises that more investigation and dialogue around the relative costs and benefits of IAP management is needed. The widespread prevalence of herbicide residues is irrefutable and evidence of risk of exposure to these compounds is growing, leading to calls for stricter herbicide regulation^2^. However, without careful comparison of chemical versus physical methods across entire lifecycles, our ability to make informed decisions around IAP management remains limited. We do note cradle-to-grave assessment of environmental impacts in this study was limited by a paucity of data on field-relevant environmental release pathways of products used for IAP management. Future effort to gather this information would be welcomed.

While wider sustainability is a vital goal for IAP management methods, the methods we employ must also be effective. For select invasive plants, including Japanese knotweed, chemical treatment is a mainstay^3,23^, though no method currently results in complete eradication. Unlike the global use of herbicides for agriculture, invasive plant management is rooted in nature conservation and sustainability^3^ and operates at smaller scales. The need for herbicides versus alternative (often untested) weed management products must therefore be informed by appropriate context and wider goals. Social perceptions of IAP management methods are an important part of this^33^ but should also be informed by empirical evidence at appropriate scale, considering the social impacts of knotweed infestation and subsequent management. From a socio-economic perspective, this study indicates that employing the most effective and sustainable approach for knotweed management is also the most cost-effective approach. Moreover, at a time where social and ethical responsibility for the environment is increasing, considering the bigger picture of IAP management methods can help prioritise these approaches. The results of this study may be used to initiate dialogue around the relative impacts and sustainability of IAP management methods and how this compares to public perceptions.

Whether the long-term impacts of plant invasions outweigh the effects and opinions of management also needs consideration to inform value- and goal-driven decisions at a strategic scale^28^. If the costs of management outweigh the benefits, efficacy, economic viability and impacts across entire lifecycles must be considered when selecting alternative treatments or appropriate mitigation methods. Integrating the impacts of invasive plants with Life Cycle Assessment of management scenarios to compare treatment with a ‘do nothing’ approach is recommended to address this. Stakeholder engagement in this matter is therefore vital if we aim to align with sustainability goals.

## Conclusions

This study assessed the environmental and economic impacts of eight management approaches for Japanese knotweed. Glyphosate foliar spray methods found to be the most effective against Japanese knotweed by Jones et al.^23^ elicited the fewest environmental and economic impacts, illustrating methods that ultimately reduced input gave better outcomes. Parsimony should therefore be an important consideration when making management decisions. Geomembrane covering imposed the greatest impacts during production and largest economic costs, followed by integrated physiochemical (excavation and herbicide application) methods. While post-treatment impacts of knotweed management methods are a current knowledge gap, evidence of the wider implications of herbicide formulations and the products and processes used in physical management methods is growing^53,57,62,76^. These results therefore underscore the need for careful (and comprehensive) consideration of risks and benefits associated with invasive plant management processes when devising invasive plant management strategies at scale^28^.

## Supplementary Information

Supplementary Information 1.

Supplementary Information 2.

Supplementary Information 3.

## Data Availability

The data generated and analysed in this study are available from the corresponding authors on reasonable request.
