# Supplementary material for: Assessing the relative impacts and economic costs of Japanese knotweed management methods
Source: Sci Rep. 2023 Mar 17;13:3872. doi: 10.1038/s41598-023-30366-9 (PMC10023688; doi:10.1038/s41598-023-30366-9)
Supplement: Supplementary file 1 — Supplementary Information 1. [file 41598_2023_30366_MOESM1_ESM.docx]

**Supplementary guide**

Supplementary file title: Supplementary Tables

Text summary: The ‘Supplementary Tables’ file contains six tables summarising application rates of herbicides assessed, materials, processes and upstream data sources used, and validated material and labour costs of each treatment as well as the numerical outputs of the comparative LCA as illustrated by Figures 2 and 4 in the main article.
