# Supplementary material for: Assessing the relative impacts and economic costs of Japanese knotweed management methods
Source: Sci Rep. 2023 Mar 17;13:3872. doi: 10.1038/s41598-023-30366-9 (PMC10023688; doi:10.1038/s41598-023-30366-9)
Supplement: Supplementary file 3 — Supplementary Information 3. [file 41598_2023_30366_MOESM3_ESM.docx]

**Supplementary information**

Supplementary Tables

**Supplementary Table 1.** Definitions of impact categories at midpoint and endpoint level used in this study, adapted from Huijbregts et al.^33^.

| **Level** | **Impact Category** | **Units** | **Description** |
| --- | --- | --- | --- |
| **Midpoint** | Global warming | kg CO_2_ equivalent (eq) | Global Warming Potential (GWP) for 100 years (data obtained from IPCC, 2013). |
|  | Stratospheric ozone depletion | kg CFC11 eq | Ozone Depletion Potential (ODP) over 100 years. |
|  | Ionizing radiation | kBq Co-60 eq | Ionizing radiation potential (IRP) relative to the emission of reference substance Cobalt-60 to air. |
|  | Ozone formation, Human health | kg NOx eq | Formation of ozone through photochemical reactions by NOx and Non-Methane Volatile Organic Compounds (NMVOCs). Human health ozone formation potential (HHOFP) calculated. |
|  | Fine particulate matter formation | kg PM2.5 eq | Particulate matter formation potential (PMFP) of fine particulate matter below 2.5 micrometers diameter. This included primary aerosols and secondary aerosols derived from SO2, NH3, NOx over 100 years. |
|  | Ozone formation, Terrestrial ecosystems | kg NOx eq | Formation of ozone through photochemical reactions by NOx and Non-Methane Volatile Organic Compounds (NMVOCs). Ecosystem ozone formation potential (EOFP) calculated. |
|  | Terrestrial acidification | kg SO_2_ eq | Acidification potential quantifies the soil acidity a substance can exhibit relative to SO2. |
|  | Freshwater eutrophication | kg P eq | Emission impacts to freshwater based on the transfer of phosphorous from soil to freshwater bodies. |
|  | Marine eutrophication | kg N eq | Marine eutrophication potential calculated as the emission of a substance scaled to the world average of N emitted to marine water. |
|  | Terrestrial ecotoxicity | kg 1,4-DCB | Toxicity potential expressed as 1 kg 1,4-dichlorobenzene equivalents. |
|  | Freshwater ecotoxicity | kg 1,4-DCB |  |
|  | Marine ecotoxicity | kg 1,4-DCB |  |
|  | Human carcinogenic toxicity | kg 1,4-DCB |  |
|  | Human noncarcinogenic toxicity | kg 1,4-DCB |  |
|  | Land use | m^2^ crop eq | Based on the relative species loss of land transformation/occupation and land relaxation |
| **Endpoint** | Damage to human health | Years | Disability-adjusted loss of life years |
|  | Damage to ecosystems | Species x year | Time-integrated species loss |
|  | Damage to resource availability | USD ($) | Surplus cost |

**Supplementary Table 2.** Application rates for each treatment of interest.

| **Treatment group ID** | **Active ingredient** | **Authorised application rate (L/g ha^-1^)** | **Authorised application rate (kg AE ha^-1^)** | **Actual application rate used (L/g ha^-1^)** | **Actual application rate used (kg AE ha^-1^)** |
| --- | --- | --- | --- | --- | --- |
| G^3.60, F, A^ | Glyphosate 360 g/L | 10 | 3.6 | 10 | 3.6 |
| G^2.16, F, S+A^ | Glyphosate 360 g/L | 6 | 4.32 | 6 | 4.32 |
| D^2.80+G2.16; F, S+A^ | 2,4-D amine 500 g/L | 2.8 | 2.8 | 4.5 | 4.5 |
|  | Glyphosate 360 g/L | 6 | 4.32 | 6 | 4.32 |
| P^2.69, F+SL, S^ + G^3.60, F, A^ | Picloram 240 g/L | 11.2 | 2.688 | 11.2 | 2.688 |
|  | Glyphosate 360 g/L | 10 | 3.6 | 10 | 3.6 |
| G^65.00, ST, A^ | Glyphosate 360 g/L | 10 | 3.6 | 180.56 | 65.002 |
| D^S^+G^3.60, F, A^ | Glyphosate 360 g/L | 10 | 3.6 | 10 | 3.6 |
| D+^P2.69, F+SL, S^ + G^3.60,F, A^ | Picloram 240 g/L | 11.2 | 2.688 | 11.2 | 2.688 |
|  | Glyphosate 360 g/L | 10 | 3.6 | 10 | 3.6 |
| Mem^COV^ | Visqueen^®^ 300 μm (1200 gauge) HDPE geomembrane | N/A | N/A | N/A | N/A |

**Supplementary Table 3.** Materials and processes used in SimaPro with upstream data sources.

| **Component** | **Material/process** | **Database** |
| --- | --- | --- |
| Glyphosate | Glyphosate (RER) at plant | Agri-footprint 5 |
| Tallow amine | Esterquat RER | Ecoinvent 3 |
| 2,4-D | 2,4-D at plant RER mass | Agri-footprint 5 |
| Picloram | Herbicide at plant/RER mass | Agri-footprint 5 |
| Diesel | 0.09 kg diesel RoW market | Ecoinvent 3 |
| Petrol | Petrol, two-stroke blend | Ecoinvent 3 |
| HDPE Geomembrane | Horticultural fleece GLO market | Ecoinvent 3 |

**Supplementary Table 4.** Cost of materials (collected from Agrigem Ltd, July 2021).

| **Product** | **Cost (£, GBP)** | **Details** |
| --- | --- | --- |
| Monsanto Amenity Glyphosate | 5.88/L | Used as a proxy for Glyfos Proactive |
| 2,4-D (Depitox^®^) | 7.20/L |  |
| Synero (£68.8/L)  Icade (£78.8/L) | 72.75/L | Average price used as proxy for Picloram (Tordon 22K^®^) |
| Visqueen^®^ 300 μm (1200 gauge) HDPE geomembrane | 48.20/100 m^2^ |  |
|  |  |  |

**Supplementary Table 5.** Data inputs for labour costs (validated by Complete Weed Control Ltd).

| **Component** | **Labour costs (£, GBP)** |
| --- | --- |
| Herbicide application and installation of geomembrane barrier | 14.02/hr |
| Machine operation | 12.87/hr |
| Use of hand tools and manual vegetation clearance | 11.70/hr |
| Excavator use (hire and labour) | 420/day |

**Supplementary Table 6.** Midpoint LCA results comparing the impacts of different Japanese knotweed treatment methods (NB – table >1 page long; see separate Excel file)

**Supplementary Table 7.** Endpoint impacts of Japanese knotweed treatment methods (see Table 1 for description of treatments).

| **Impact category** | **Unit** | **G^3.60, F, A^** | **G^2.16, F, S+A^** | **D^2.80^+G^2.16; F, S+A^** | **P^2.69, F+Sl, S^ + G^3.60,^ ^F, A^** | **P^2.69, F+Sl, S^ + G^3.60,^ ^F, A(proj)^** | **G^65.00, St, A^** | **D^S^+G^3.60, F, A^** | **D+P^2.69, F+Sl, S^ + G^3.60,F, A^** | **D+P^2.69, F+Sl, S^ + G^3.60,F, A(proj)^** | **Mem^Cov^** |
| --- | --- | --- | --- | --- | --- | --- | --- | --- | --- | --- | --- |
| Human health | DALY | 5.16E-04 | 5.63E-04 | 1.13E-03 | 1.07E-03 | 1.15E-03 | 2.17E-03 | 2.95E-03 | 3.58E-03 | 3.73E-03 | 2.92E-03 |
| Ecosystems | Species.yr | 2.27E-06 | 2.47E-06 | 3.84E-06 | 3.64E-06 | 3.89E-06 | 9.51E-06 | 7.77E-06 | 9.26E-06 | 9.66E-06 | 7.34E-06 |
| Resource use | USD | 1.66E+01 | 1.81E+01 | 4.40E+01 | 1.40E+02 | 1.44E+02 | 6.97E+01 | 8.30E+02 | 8.63E+02 | 8.70E+02 | 2.30E+02 |
| Cost of impacts | GBP | 5.13E+01 | 5.59E+01 | 1.18E+02 | 1.86E+02 | 1.95E+02 | 2.15E+02 | 8.44E+02 | 9.16E+02 | 9.33E+02 | 3.92E+02 |
| Cost to implement | GBP | 1.57E+03 | 3.22E+03 | 2.98E+03 | 5.67E+03 | 7.21E+03 | 3.44E+03 | 8.41E+03 | 1.10E+04 | 1.18E+04 | 4.78E+04 |
| Total economic cost | GBP | 1.62E+03 | 3.28E+03 | 3.10E+03 | 5.86E+03 | 7.40E+03 | 3.66E+03 | 9.25E+03 | 1.20E+04 | 1.28E+04 | 4.82E+04 |
